# Effects of Intestinal Microorganisms on Metabolism and Toxicity Mitigation of Zearalenone in Broilers

**DOI:** 10.3390/ani12151962

**Published:** 2022-08-02

**Authors:** Sifan Jia, Chenxi Ren, Ping Yang, Desheng Qi

**Affiliations:** Department of Animal Nutrition and Feed Science, College of Animal Science and Technology, Huazhong Agricultural University, No.1, Shizishan Street, Wuhan 430070, China; jiasifan@webmail.hzau.edu.cn (S.J.); rcx7@webmail.hzau.edu.cn (C.R.); yp923418@webmail.hzau.edu.cn (P.Y.)

**Keywords:** zearalenone, broiler, metabolites, intestinal microbe, hepatotoxicity

## Abstract

**Simple Summary:**

Zearalenone (ZEN) widely contaminates all the feed crops, and ZEN may cause harmful damage to animals and humans. Different animals have different sensitivity to ZEN. Among these animals, chickens show a strong resistance. Intestinal microorganisms are essential in digestion and degradation. Therefore, we hypothesise whether intestinal microorganisms in chickens play an important role in digesting and degrading ZEN. In this study, we found that intestinal microorganisms could degrade ZEN to a certain degree by both vivo and vitro experiments. We concluded that the intestinal microbiota of broilers had metabolic effects on ZEN and alleviated antioxidant and liver damage caused by ZEN to broilers. Moreover, we found some key bacteria that are important in degrading ZEN.

**Abstract:**

Zearalenone (ZEN) is an estrogenic mycotoxin, and chickens are relatively insensitive to it. In this study, the effects of intestinal microorganisms on ZEN metabolism and toxicity mitigation in broilers were studied by two experiments. Firstly, in vitro, ZEN was incubated anaerobically with chyme from each part of the chicken intestine to study its intestinal microbial metabolism. Then, in vivo, we explored the effects of intestinal microbiota on ZEN by inhibiting intestinal microorganisms. Broilers were fed a control diet, 2.5 mg/kg ZEN diet, microbial inhibition diet or ‘microbial inhibition +2.5 mg/kg ZEN’ diet. In vitro, the results showed that the rates of ZEN degradation by microorganisms in the duodenum, ileum, caecum, and colon were 56%, 12%, 15%, and 17%, respectively, and the microorganisms could convert ZEN into Zearalenol (ZOL). After microbial inhibition in vivo, the content of ZEN and its metabolites in excreta of broilers increased significantly, and antioxidant damage and liver damage were aggravated. 16S rRNA sequencing results showed that antioxidant indices and the content of ZEN and its metabolites in excreta were significantly correlated with the relative abundance of *Streptococcus, Lactococcus* and *Enterococcus*, etc. In conclusion, the intestinal microorganisms of broilers play an important role in ZEN metabolism and ZEN-induced antioxidant and liver injury mitigation, among which the key bacteria include *Streptococcus*, *Lactococcus* and *Enterococcus*, etc.

## 1. Introduction

Zearalenone (ZEN) is one of the most common mycotoxins produced mainly by *Fusarium graminearum* and widely contaminates corn, wheat, barley, sorghum, rice, and other grains [1,2,3]. ZEN and its metabolites have a biological structure similar to natural oestrogen β-oestradiol and could bind to estrogen receptors in animals. ZEN affect the chromosome structure, and related gene transcription and translation, as well as interference in the expression of estrogen and organ function, cause hypoestrogenism in animals [4,5,6]. Furthermore, ZEN has reproductive toxicity, liver and kidney toxicity, and immunotoxicity [1,7,8]. It could reduce the nutritional value of feed, damage the growth and health of livestock and poultry, and cause huge economic losses to livestock production. 

There are apparent species differences in the sensitivity of animals to ZEN. Pigs are very sensitive to ZEN [7,9,10], whereas poultry has a relatively stronger tolerance. Diets containing 0, 50 and 200 mg/kg ZEN affected some physiological and biochemical indices of laying hens and turkeys, but showed no effect on reproductive function [11,12]. A previous study showed that 2 mg/kg ZEN could cause achondroplasia and reduce growth performance in broilers [13], and 7.9 mg/kg ZEN in broiler diet could increase the activity of γ-glutamyl transferase in plasma, increase the activity of glutathione peroxidase in kidney, and cause oxidative stress [14].

There are many reasons for chicken’s insensitivity to ZEN [15,16,17], and intestinal microbes may be an important reason [18]. ZEN is mainly metabolized in animals through reduction and binding reaction and could be biotransformed by liver and intestinal microbes. ZEN is degraded to two isomers, α and β-zearalenol (α/β-ZOL) [15,19,20,21]. The reaction producing α-ZOL could be considered as the toxicity enhancement pathway and the reaction producing β -ZOL as a detoxification pathway [22,23]. 

In this study, the anaerobic incubation of intestinal contents and ZEN in vitro was adopted to evaluate and quantify the capacity of intestinal microorganisms to degrade and metabolize ZEN [24]; moreover, antibiotic-induced dysbiosis of the gut microbiota in vivo was used to infer the role of microorganisms [25]. Then, 16S rRNA sequencing analysis was used to clarify the changes of the intestinal microbiota and predict the key microbiota related to ZEN metabolism. The results of this study could provide a reference for future research on ZEN detoxification technology and the rational utilization of feed materials. 

## 2. Materials and Methods

### 2.1. Analytes and Reagents

The analytical standards of ZEN, α-ZOL, and β-ZOL, used for both in vitro and the animal experiment, was obtained from Sigma-Aldrich, CA, USA. Standards were diluted in acetonitrile to obtain working solutions of ZEN, α-ZOL, and β-ZOL for assays with High Performance Liquid Chromatography (HPLC) and Ultra Performance Liquid Chromatography Tandem Mass Spectrometry (UPLC). All analytical standards were stored at ≤−15 °C. The internal standard (IS) --^13^C_18_-ZEN, enzyme of β-glucuronidase used for hydrolysis of the conjugate, and QuECHERs used for impurities were purchased from Sigma, CA, USA. Chemicals of chloroform and ethyl acetate, used for ZEN extraction, were purchased from Sinopharm Chemical Reagent, Shanghai, China.

### 2.2. In Vitro Test

#### 2.2.1. Anaerobic Incubation of ZEN with the Microflora

Duodenal, jejunal, ileal, caecal, and colonic chyme were collected from 42-day-old broilers as samples (*n* = 6). Samples were weighed and diluted to a final concentration of 10% (*W*/*V*) with PBS (Gibco, New York, NY, USA). Then, 10% of intestinal slurry and 6 μg/mL ZEN were mixted with PBS 1:1 (the final concentration was 5% intestinal slurry with 3 μg/mL ZEN) under anaerobic conditions, then incubated anaerobically at 39 °C for 6 h. In the control group, cadmium chloride (10 mg/mL) (Sinopharm Chemical Reagent, Shanghai, China) was added to 10% sample slurries to inactivate the microorganisms for 30 min before the contents were incubated with ZEN. After the incubation, pre-cooled methanol was added to terminate the reaction, and the supernatant liquors were taken for testing.

#### 2.2.2. Analysis of ZEN and its Metabolites

QuECHERs reagent was added to supernatant liquors from section 4.1.1 [26], and, after vortexing, the supernatant liquors were taken, extracted with chloroform three times [27] (in clean bench) and dried under nitrogen at 60 °C. The ZEN and its metabolites were analysed by HPLC, according to Videmann et al. [28] and Koraichi et al. [29] with modifications. The Agilent 1260 HPLC system (Agilent, San Jose, CA, USA) used was equipped with a UV detector, quaternary high-pressure pump, and an Agilent TC-C18 reversed-phase column (3.5 μm, 4.6 mm × 150 mm, San Jose, CA, USA). UV detection wavelength: 270 nm. Mobile phase: methanol:acetonitrile:water = 50:15:35. Flow rate: 1.0 mL/min. Column temperature: 30 °C. The limit of detections of ZEN and its metabolites for HPLC system was 0.01 μg/mL.

### 2.3. Animal Experiment

#### 2.3.1. Animal

A total of 120 1-day-old broilers (half female and half male) were randomly divided into four groups with 6 replicates per group: control, 2.5 mg/kg ZEN, microbial inhibition, and microbial inhibition +2.5 mg/kg ZEN. The two microbial inhibition groups received a basic diet containing streptomycin (1 g/kg, Biosharp, Shanghai, China), ampicillin (1 g/kg, Biosharp, Shanghai, China), and neomycin (1 g/kg, Yuanye, Shanghai, China) to clear intestinal bacteria [30]. ZEN and antibiotics were mixed in powdery feed using a stepwise dilution method. During the entire 3-week experimental period, broilers were reared in three-layer cages, the temperature was adjusted using a warm lamp, and water was supplied to the broiler chickens ad libitum. Ethical guidelines for animal protection rights were observed.

#### 2.3.2. Sample Collection

From day 10 to day 21, the fresh excreta in each cage on the day were collected every day, dried crushed and mixed together, and stored at −20 ℃ for testing. Twelve broilers were randomly selected from each group; their venous blood was kept at room temperature until the blood coagulated, and the serum obtained after centrifugation at 3500 r/min for 10 min was stored at −80 °C until use. The broilers were stunned by electrocution and killed by exsanguination. The contents of the duodenum, ileum, and caecum were collected in cryopreservation tubes for microbial diversity analysis. The middle part of the liver was collected in a cryopreservation tube. The middle part of the liver of four broilers in each group was randomly collected and fixed in 4% paraformaldehyde for making paraffin sections for histopathological examination.

#### 2.3.3. DNA Extraction and PCR Amplification

Microbial community genomic DNA was extracted using an E.Z.N.A.® Soil DNA Kit (Omega Bio-Tek, Norcross, GA, USA) according to the manufacturer’s instructions. The DNA extract was checked on 1% agarose gel, and DNA concentration and purity were determined with a NanoDrop 2000 UV-vis spectrophotometer (Thermo Scientific, Wilmington, DE, USA). The hypervariable V3–V4 region of the bacterial 16S rRNA gene was amplified with primer pairs 338F (5′-ACTCCTACGGGAGGCAGCAG-3′) and 806R (5′-GGACTACHVGGGTWTCTAAT-3′) with an ABI GeneAmp® 9700 PCR thermocycler (ABI, CA, USA). PCR amplification of the 16S rRNA gene was performed as follows: initial denaturation at 95 °C for 3 min, followed by 27 cycles of denaturing at 95 °C for 30 s, annealing at 55 °C for 30 s and extension at 72 °C for 45 s, then, single extension at 72 °C for 10 min and ending at 4 °C. PCR reactions were performed in triplicate. The PCR product was extracted from 2% agarose gel and purified using an AxyPrep DNA Gel Extraction Kit (Axygen Biosciences, Union City, CA, USA) according to the manufacturer’s instructions and quantified using a Quantus™ Fluorometer (Bellefonte, PA, USA).

#### 2.3.4. Illumina MiSeq Sequencing

Purified amplicons were pooled in equimolar concentration and paired-end sequenced on an Illumina MiSeq PE300 platform/NovaSeq PE250 platform (Illumina, San Diego, CA, USA) according to the standard protocols of Majorbio Bio-Pharm Technology Co. Ltd. (Shanghai, China). The raw reads were deposited into the NCBI Sequence Read Archive (SRA) database [31].

#### 2.3.5. Processing of Sequencing Data

The raw 16S rRNA gene sequencing reads were demultiplexed, quality-filtered by fastp version 0.20.0 [32] and merged using FLASH version 1.2.7. Operational taxonomic units (OTUs) with 97% similarity cut-off [33,34] were clustered using UPARSE version 7.1, and chimeric sequences were identified and removed. The taxonomy of each OTU representative sequence was analyzed by RDP Classifier version 2.2 [35] against the 16S rRNA database using a confidence threshold of 0.7 [36,37].

#### 2.3.6. ZEN and its Metabolites of Excreta Analysis

Excremental samples +5 mL of ammonium acetate buffer +40 μL of β-glucuronidase solution were shaken overnight at 37 °C in an air bath shaker. The enzymatically hydrolysed sample +6 mL of 0.1% acetonitrile acetic acid was extracted by shaking, and then 1 g of NaCl was added. After vortex centrifugation, 4 mL of the supernatant solution was taken, and QuECHERs reagent was added for centrifugation. The result was reconstituted with ultrapure water, then extracted with chloroform and ethyl acetate. The collected supernatant was blown dry with nitrogen, and the residue was dissolved in 300 μL of acetonitrile/water and passed through a 0.22 μm nylon filter for analysis.

Considering that impurities in excreta are difficult to purify, Liquid Chromatograph Mass Spectrometer (LC-MS) analysis was adopted. An API5000 UPLC-MS system was equipped with a Hypersil GOLD column (5 μm, 150 × 2.1 mm) (ThermoFisher, Bellefonte, PA, USA) set at 40 °C; 0.1% acetic acid water (A) and acetonitrile (B) were used as eluents at a flow rate of 0.3 mL/min with the following gradient profile: 0–0.5 min (70% A, 30% B), 0.5–5.0 min (70% B), 5.0–6.4 min (30% A, 70% B), 6.4–6.5 min (30% B), and 6.5–8.5 min (70% A, 30% B) [17,38]. Per run, 10 μL of sample was injected. The limit of detections of ZEN and its metabolites for UPLC-MS system is 5 × 10^−4^ μg/mL.

Mass spectrometry conditions: electrospray ionization (ESI) negative ion mode; multiple reaction detection (MRM); electrospray voltage 4500 V; ion source temperature 250 °C; atomization and desolvation gas: nitrogen; gas flow: 10 L/minute; nebulizer: 25 psi [39]. Mass spectrum parameters are shown in Table 1.

#### 2.3.7. Antioxidant Analysis

Serum total antioxidant capacity (T-AOC), total superoxide dismutase (T-SOD), liver malondialdehyde (MDA), glutathione (GSH), and SOD were determined using an ELISA kit (Cloud-Clone Corp., Houston, TX, USA). The specific details were in kit instructions. In brief:

For T-AOC, T-SOD, and SOD, prepare all reagents, samples and standards, add 100 μL standard or sample to each well and incubate 1 hour at 37 °C; aspirate and add 100 μL prepared detection reagent A, and incubate 1 hour at 37 °C, aspirate and wash 3 times; add 100 μL prepared detection reagent B and incubate 30 mins at 37 °C, aspirate and wash 5 times; add 90 μL substrate solution and incubate 10–20 minutes ate 37 °C; add 50 μL stop solution and read at 450 nm immediately.

For MDA and GSH, prepare all reagents, samples and standards, add 50 μL standard or sample to each well and then, add 50 μL prepared detection reagent A immediately, shake and mix, incubate 1 hour at 37 °C; aspirate and wash 3 times; add 100 μL prepared detection reagent B and incubate 30 mins at 37 °C, aspirate and wash 5 times; add 90 μL substrate solution and incubate 10–20 minutes ate 37 °C; add 50 μL stop solution and read at 450 nm immediately.

#### 2.3.8. Histopathology

After embedding in paraffin blocks, formalin-fixed liver tissues were sectioned to 3–5 μm thicknesses on a microtome. The sections were placed on silicon-coated glass slides, dried, deparaffinized with xylene and rehydrated in decreasing ethanol series. After the sections were cleaned with distilled water, dyed in hematoxylin solution for 6–7 min, and stained in eosin for 90 s, they were dehydrated with high-concentration ethanol for a few minutes and then passed through xylene to make the sections transparent [40]. Neutral gum was placed on a glass slide, covered with a cover glass and, after the gum solidified, it was observed under an upright fluorescence microscope (Olympus BX53, Osaka, Japan).

### 2.4. Statistical Analysis

Statistical analysis was performed using SPSS (version 20). Using the general linear model procedure (GLM) in the software, double factor variance analysis and Wilcoxon rank–sum test were performed. If there was a significant main effect or interaction effect, Duncan’s multiple comparisons were used for post hoc testing, and Student’s *t*-test was used to analyse the differences between the two treatment groups. The results are expressed as mean ± SD; *p* < 0.05 means significant difference, *p* < 0.01 means an extremely significant difference. Graphs were drawn using GraphPad Prism 6.0.

## 3. Results

### 3.1. Effect of In Vitro Incubation with Chicken Gut Microbiota on ZEN

The ZEN degradation rate was calculated based on the control group without microbial activity (Table 2). The results showed that the jejunum had no significant degradation effect on ZEN, whereas the microorganisms in the duodenum, ileum, caecum, and colon degraded 56%, 12%, 15%, and 17% of the ZEN, respectively. ZEN was metabolized to ZOL by microorganisms in the duodenum, caecum, and colon. Approximately equal conversion to α-ZOL and β-ZOL occurred in duodenum, while about twice as much β-ZOL was found in colon.

### 3.2. ZEN and Microbial Inhibition Alter the Gut Microbiota In Vivo

#### 3.2.1. Beta Diversity Analysis

**Figure 1:**
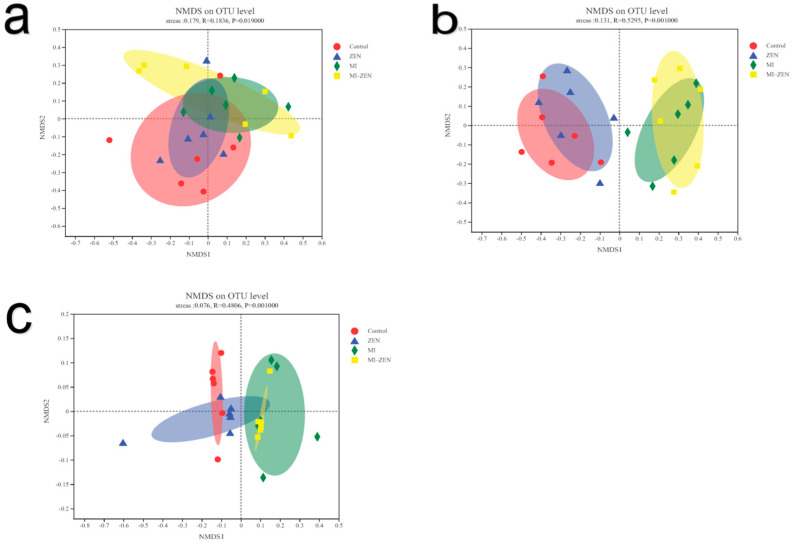
Beta diversity analysis of duodenum (**a**), ileum (**b**), and caecum (**c**). Bacteria after ZEN Treatment and Microbial Inhibition (*n* = 6). The different groups are shown on the right of the chart. The distance between points in the sample indicates the difference in species composition between samples. Horizontal and vertical coordinates are meaningless.

Beta diversity is an indicator that characterizes the similarity of microbial composition between groups. Different treatment groups were analyzed based on bray–curtis distance, and non-parametric ANOSIM analysis was used to test whether the differences between groups were significant. As Figure 1 shows, the duodenum (*p* = 0.019), ileum (*p* = 0.001), and cecum (*p* = 0.001) demonstrated a notable separation of two microbial inhibition groups from the control and ZEN groups (*p* < 0.05). Taken together, microbial inhibition significantly altered broiler gut microbiota.

#### 3.2.2. ZEN Alter the Bacterial Abundance

**Figure 2:**
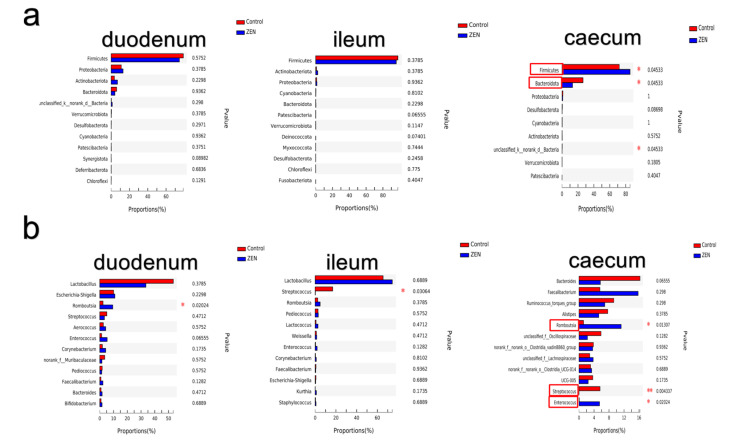
Relative abundance of duodenum, ileum and caecum microbiota between control and ZEN groups (*n* = 6). Variations in the relative abundance of duodenum, ileum and caecum microbiota at the phylum level (**a**). Variations in the relative abundance of duodenum, ileum and caecum microbiota at the genus level (**b**). * indicated *p* < 0.05, ** indicated *p* < 0.01. Data were analysed using Wilcoxon rank–sum test.

As Figure 2 shows, *Firmicutes* (72.8%) and *Proteobacteria* (16.4%) were the predominant bacteria at the phylum level in the duodenum (Figure 2a). *Firmicutes* (97%) was the predominant phylum in the ileum. *Firmicutes* (71.9%) and *Bacteroidota* (16.4%) were the predominant bacteria at the phylum level in the caecum. ZEN significantly increased the relative abundance of *Firmicutes* and significantly decreased the relative abundance of *Bacteroidota* in the caecum. At the genus level, *Lactobacillus* was the predominant bacterium in the duodenum and ileum (Figure 2b). ZEN significantly increased the relative abundance of *Romboutsia* in the duodenum and caecum (Figure 2b) and of *Enterococcus* in the caecum, and it significantly decreased the relative abundance of *Streptococcus* in the ileum and caecum.

#### 3.2.3. Microbial Inhibition Alter the Gut Microbiota

**Figure 3:**
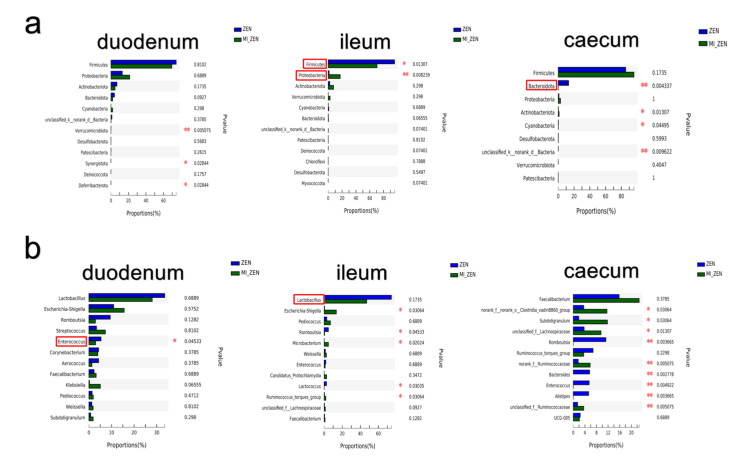
Relative abundance of duodenum, ileum and caecum microbiota between ZEN and microbial inhibition + ZEN (MI+ZEN) Groups (*n* = 6). Variations in the relative abundance of duodenum, ileum and caecum microbiota at the phylum level (**a**). Variations in the relative abundance of duodenum, ileum and caecum microbiota at the genus level (**b**). * indicated *p* < 0.05, and ** indicated *p* < 0.01. Data were analysed using Wilcoxon rank–sum test.

Figure 3 shows a clear difference between the gut microbiota of broilers in ZEN and microbial inhibition +ZEN (MI+ZEN) groups. Notably, microbial inhibition significantly decreased the relative abundance of *Firmicutes* and significantly increased the relative abundance of *Proteobacteria* at the phylum level in the ileum (Figure 3a), and *Actinomycetes* was the third dominant phylum in the ileum. Microbial inhibition significantly decreased the relative abundance of *Bacteroidota* in the caecum (Figure 3a). Then, we analysed the composition of gut microbiota at the genus level. After microbial inhibition, the relative abundance of *Enterococcus* was decreased significantly in the duodenum and caecum (Figure 3b). The relative abundance of *Lactococcus* was decreased significantly in the ileum (Figure 3b). Microbial inhibition significantly increased the relative abundance of some *Ruminococcus* species.

### 3.3. Metabolism of ZEN in Broilers

**Figure 4:**
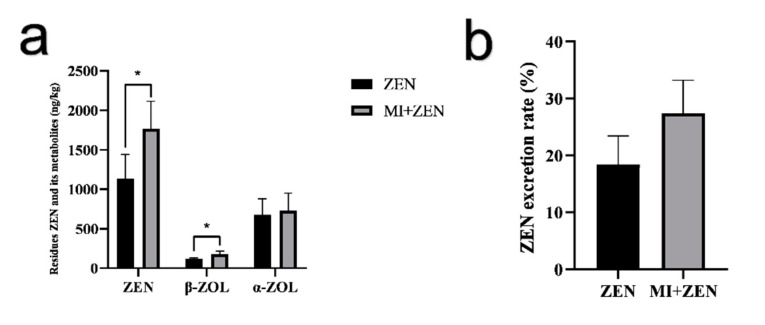
Residues of zearalenone and its metabolites in excreta of broilers (**a**) and excretion rate of zearalenone (**b**). Data represent mean values of experiments conducted in duplicate (*n* = 4) and based on HPLC-MS analyses. * indicated *p* < 0.05. Acid-insoluble ash (AIA) used as an indicator to determine the digestibility of ZEN in broilers (*n* = 4). Excretion Rate = [(Mycotoxin-e/Mycotoxin-f) × (AIAf/AIAe)] × 100 where Mycotoxin-e (ZEN and its metabolites) and AIAe are the concentrations of mycotoxin components and of AIA in the excreta, respectively, and Mycotoxin-f (ZEN and its metabolites), and AIAf represent the concentrations of the same mycotoxin components and AIA in the feeds.

As Figure 4 shows, ZEN was mainly reduced to α-ZOL in broilers, and the content of α-ZOL was about 6 times that of β-ZOL. Compared with the ZEN group, the ZEN and β-ZOL content in excreta in the MI+ZEN group was increased significantly (*p* < 0.05) by 56.1% and 54%, respectively. Moreover, total content of ZEN and its metabolites was increased by 39%. As shown in Figure 4b, the excretion rate in ZEN and MI+ZEN groups were 18.5% and 27.5%, respectively. ZEN excretion rate was about 9% higher in the MI+ZEN group.

### 3.4. Oxidative Stress and Liver Injury

**Figure 5:**
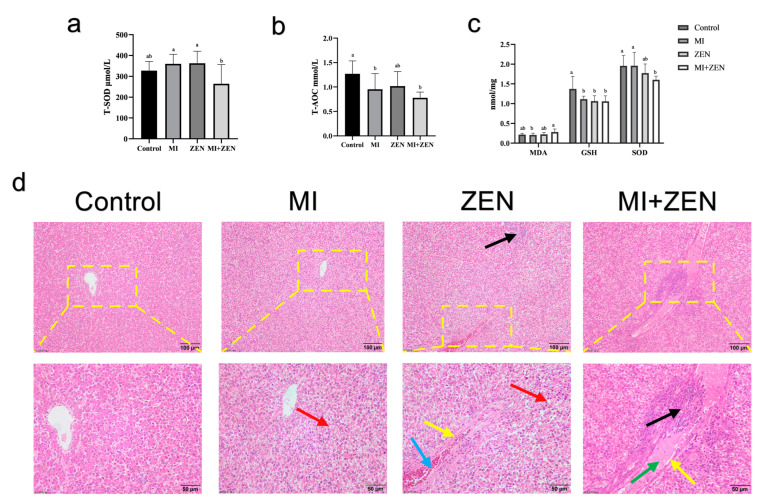
Effect of ZEN and microbial inhibitor administration on oxidative and liver damage in broilers. Data represent mean ± SD. Serum T-SOD (**a**) and T-AOC (**b**) level (*n* = 8). Liver oxidative index (**c**), including MDA, GSH, and SOD level (*n* = 6). Mean values without a common letter (a, b) were significantly different (*p* < 0.05). (**d**) Representative images of H&E-stained liver sections (magnification ×200 and ×400). Black arrows indicate infiltration of inflammatory cells; red arrows indicate tiny, circular vacuoles; blue arrows indicate vascular congestion; green arrows indicate protein mucus; and yellow arrows indicate perivascular oedema.

Figure 5 shows that, compared with the ZEN group, the serum T-SOD (total superoxide dismutase) content in MI+ZEN group was significantly decreased (Figure 5a). Compared with the control group, the T-AOC (total antioxidant capacity) level of the MI+ZEN group decreased significantly (Figure 5b). ZEN at 2.5 mg/kg significantly decreased the activity of GSH (glutathione) in the liver (*p* < 0.05). Compared with the control group, GSH activity and SOD (superoxide dismutase) content in the MI+ZEN group were significantly decreased (*p* < 0.05). As shown in Figure 5d, the liver tissues of the ZEN group showed some pathological changes, including obvious infiltration of inflammatory cells in liver tissues, peripheral oedema of blood vessels, vascular congestion in blood vessels, and slight circular vacuolation of cells. However, in the MI+ZEN group, a large amount of protein mucus appeared in liver tissue and inside blood vessels, and severe inflammatory cell infiltration occurred around blood vessels, as well as peripheral vascular oedema and slight circular vacuolation of stem cells.

### 3.5. Spearman Correlation Heatmap

**Figure 6:**
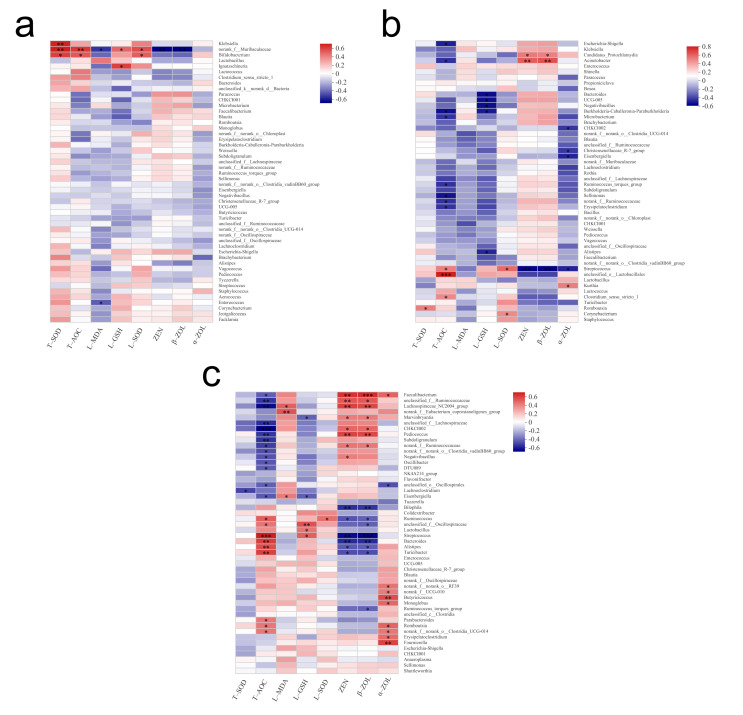
Correlation analysis of duodenum (**a**), ileum (**b**) and caecum (**c**) Microbiota with oxidative index and metabolism of ZEN. Data information in two-dimensional matrix or table is reflected by color changes. Color depth represents the size of data value. It intuitively expresses the size of data value by defined color depth. The X-axis and Y-axis are clinical factors and species, respectively, and the correlation R value and P value were obtained through calculation. The R value is displayed in different colors in the figure. * indicated *p* < 0.05, ** indicated *p* < 0.01, *** indicated *p* < 0.001.

A correlation heatmap analysis was conducted by calculating correlation coefficients between clinical factors and selected species; the obtained numerical matrices are presented visually through the heatmap. According to the correlation analysis of duodenal microbiota (Figure 6a), the relative abundance of *norank_f_Muribaculaceae* was significantly positively correlated with the antioxidant capacity and significantly negatively correlated with the content of ZEN and β-ZOL in excreta (*p* < 0.01). As shown in Figure 6b, T-AOC had a significant positive correlation with *unclassified_o_Lactobacillales* (*p* < 0.001). *Streptococcus* was positively correlated with T-AOC and SOD content in the liver (*p* < 0.05) and significantly negatively correlated with ZEN and α/β-ZOL content in excreta (*p* < 0.01). According to the correlation analysis of caecal microbiota (Figure 6c), *Streptococcus* was significantly positively correlated with T-AOC and liver GSH content and significantly negatively correlated with ZEN and β-ZOL content in excreta. The content of ZEN and its metabolites in excreta was significantly positively correlated with *Faecalibacterium* and *Ruminococcaceae* and negatively correlated with *Bacteroides*, *Alistipes*, and *Bilophila*.

## 4. Discussion

The gastrointestinal tract is an important filter against mycotoxins, and gut microbiome, which includes bacteria, viruses, and fungi, is considered a "microbial organ" and comprises >100 trillion microbial cells [41]. Host phenotype is regulated by its genes and influenced by "host second genome"–gut microbiome. Gut microbiota and its metabolites influence immune diseases, nutrient metabolism, and animal behavior through gut–liver and gut–brain axes [42]. When ZEN is absorbed by animals, the gastrointestinal system is the first immune organ, and gut microbes are thought to be key to maintaining the intestinal barrier [15,43,44]. In the 1980s, the important influence of gut microbes on mycotoxin toxicity was demonstrated in ruminants [45]. It has been proved that intestinal microorganisms play an important role in the metabolism and biotransformation of ZEN [44,46,47], and many microbial strains that could adsorb and metabolize ZEN have been isolated from animal and human intestines [48,49,50]. Mendez-Catala et al. [24] developed an in vitro model to quantify the intestinal microbial bioactivation and detoxification of zearalenone. Their results showed that intestinal microorganisms degrade ZEN and produce α/β-ZOL under anaerobic conditions, and its activity may be 36% of liver activity. However, there have been no tests to prove the effects of chicken gut microbes on ZEN so far. In this study, the duodenum, jejunum, ileum, cecum, and colonic chyme of chickens were co-incubated with ZEN under anaerobic conditions in vitro to analyze the degradation and transformation of ZEN by intestinal microorganisms. Then, we fed purified ZEN to chickens and disturbed the intestinal microorganisms with antibiotics, detected the changes in the relative abundance of intestinal microbes, analysed the differences in metabolism of ZEN after microbial inhibition, and explored whether the intestinal microorganisms alleviated the harm of antioxidant and liver damage caused by ZEN to chickens.

The in vitro study allowed us to fiund that microorganisms in duodenum, ileum, cecum, and colon could degrade ZEN, with the highest degradation rate of 56% being in duodenum. This may be due to differences in the dominant species in each intestine. In addition, the conversion rate of ZEN to chicken intestinal microbes in vitro was only 1–4%, and more β -ZOL was produced in cecum. It was reported that pig intestinal suspensions were incubated with ZEN under anaerobic conditions, and ZEN was degraded by microorganisms in the cecum and colon at a rate of about 12% and bioconverted to α -ZOL, whereas in the small intestine it was not [51]. Some studies have shown that the difference in absorption is mainly related to the distribution of small intestinal microorganisms [52,53]. Moreover, pigs have very few bacteria in their small intestines compared to chickens. ZEN is absorbed in the proximal small intestine [54,55], but in gilts, ZEN was not degraded by small intestine microorganisms [51,56].

We found that the dominant bacterium in the duodenum and ileum of broilers are *Lactobacillus* based on 16S rRNA sequencing technology. There are reports that one of the most promising types of organism able to neutralize ZEN seems to be lactic acid bacteria [57], and *Lactobacillus* has the ability to bind and biotransform ZEN [27,58,59]. Whether *Lactobacillus* in the chicken gut could detoxify ZEN remains to be studied. Niderkorn et al. found that *Streptococcus* and *Enterococcus* could bind more ZEN than other genera to reduce toxicity to animals [50]. Similarly, we found that ZEN significantly changed the relative abundance of *Streptococcus* and *Enterococcus* in the ileum and caecum, which may play a role in ZEN’s detoxification mechanism. After microbial inhibition, the relative abundance of *Firmicutes* in ileum was significantly decreased compared with the ZEN group, and the relative abundance of Bacteroidetes in the caecum was also significantly decreased compared with the ZEN group. Studies have shown that the ratio of *Bacteroidetes* to *Firmicutes* in the gut affects the ability to absorb substances [60]. There were significant differences in the relative abundance of several intestinal bacteria, including beneficial bacteria such as *Enterococcus*, *Lactococcus*, and *Ruminococcus*. Then, we detected the content of ZEN and its metabolites in excreta of the ZEN and MI+ZEN groups. Different from the in vitro test, broiler chickens convert ZEN to α-ZOL more. Metabolism in chicken is a complex process, and some studies have shown that the liver of broiler chickens could convert ZEN to α -ZOL more [61]. We found that the content of ZEN and β-ZOL significantly increased after microbial inhibition, which may be due to the ability of intestinal microbes to adsorb and degrade ZEN. However, the α -ZOL content did not change; we speculated that most of the biotransformation of α-ZOL in broilers might be other organs such as liver, and the scientific significance of intestinal microbiome detoxification in broilers needs further study. In addition, studies have shown that intestinal microbiome disorders are closely related to the occurrence of inflammation and injury in animals [62,63], and intestinal microbiome also plays an important protective role in mycotoxin-induced liver inflammation [30]. Studies have shown that ZEN could produce excessive ROS in animals, consume SOD, and GSH, thus reducing T-AOC in animals. Zhu et al. showed that 2 mg/kg ZEN could affect serum metabolite levels of broilers and cause liver oxidative damage [64]. In the MI+ZEN group, we found that the serum antioxidant index T-AOC decreased significantly and its concentration was the lowest, the liver antioxidant indices GSH activity and T-SOD content decreased significantly, and MDA content showed an upward trend (MDA is an important parameter reflecting the potential antioxidant capacity). Moreover, a large amount of protein mucus, peripheral vascular oedema, and severe inflammatory cell infiltration appeared in liver tissue and blood vessels, and the liver cells displayed round vacuolation. These results indicate that intestinal microbes have a protective effect in broilers and alleviate antioxidant and liver damage caused by ZEN.

Then, we further analysed the correlation between intestinal microbes and antioxidant indices and ZEN metabolism of broilers based on 16S rRNA sequencing data. The content of ZEN and its metabolites in excreta was significantly negatively correlated with the relative abundance of *Muribaculaceae*, *Streptococcus*, *Bacteroides*, *Alistipes*, and *Bilophila*. It had a significant positive correlation with *Faecalibacterium* and a partial one with *Ruminococcaceae*. Studies have shown that *Muribaculaceae* plays a role in intestinal mucin degradation, and the presence of gastrointestinal mucus reduces the ability of probiotics to bind mycotoxins [62,65]. *Bacteroides*, *Alistipes*, and *Bilophila* are biliary resistant strains, which are related to bile emission. As a component of enterohepatic circulation, bile plays an important role in poultry excretion of ZEN and its metabolites [66,67]. Furthermore, Reddy et al. indicated that *Faecalibacterium* and *Ruminococcaceae* may play an important role in ZEN’s metabolism [48]. Therefore, we speculated that the significant difference in ZEN metabolism after microbial inhibition might be related to the above strains. However, their specific functions in chicken gut microbiome needs further study.

## 5. Conclusions

In conclusion, based on the results of in vitro and in vivo experiments, we concluded that the intestinal microbiota of broilers could degrade and metabolize ZEN and alleviated the antioxidant and liver damage caused by ZEN to broilers. We found that *Lactobacillus*, *Streptococcus*, *Ruminococcus*, and *Enterococcus* of broilers play an important role in ZEN metabolism. However, further studies are needed to explore the degradation mechanism of probiotic ZEN detoxification in broilers and the types of chemical interactions involved in the binding mechanism, and the scientific basis of microbial detoxification in broilers.

## Figures and Tables

**Table 1:** Mass spectrum parameters for zearalenone and its metabolites.

Analyte	Retention Time (min)	Molecular Weight	Parent Ion (m/z)	Product ion (m/z)	Collision Energy (eV)	Crushing Voltage (V)
ZEN	6.9	318.36	317	174.6 130.6	20 25	135
α-ZOL	6.1	320.38	319.1	275.1 159.8	15 25	135
β-ZOL	5.5	320.38	319.1	275.1 159.8	15 25	105

**Table 2:** Results of the Anaerobic Incubation of Zearalenone with Microflora of Different Bowel Segments.

Intestinal Contents	ZEN Degradation Rate (%)	Conversion Rate (%)	
		α-ZOL	β-ZOL
Duodenum	55.7 ± 4.6	1.6 ± 0.6	1.8 ± 1.0
Jejunum	0	/	/
Ileum	11.9 ± 5.6	/	/
Cecum	15.1 ± 5.9	1.1 ± 0.3	/
Colon	16.6 ± 9.8	1.7 ± 0.6	3.4 ± 2.2

Note: Values are presented as means ± SD (*n* = 6). “/” indicates not detected.

## Data Availability

Raw data are stored in private computers, property of Huazhong Agricultural University, and are available upon request.
